# On-Chip Enrichment System for Digital Bioassay Based on Aqueous Two-Phase System

**DOI:** 10.1021/acsnano.2c06007

**Published:** 2022-12-29

**Authors:** Yoshihiro Minagawa, Shoki Nakata, Motoki Date, Yutaro Ii, Hiroyuki Noji

**Affiliations:** † Department of Applied Chemistry, The University of Tokyo, 7-3-1 Hongo, Bunkyo-ku, 113-8656, Japan

**Keywords:** digital bioassay, ATPS, LLPS, enrichment, microreactors

## Abstract

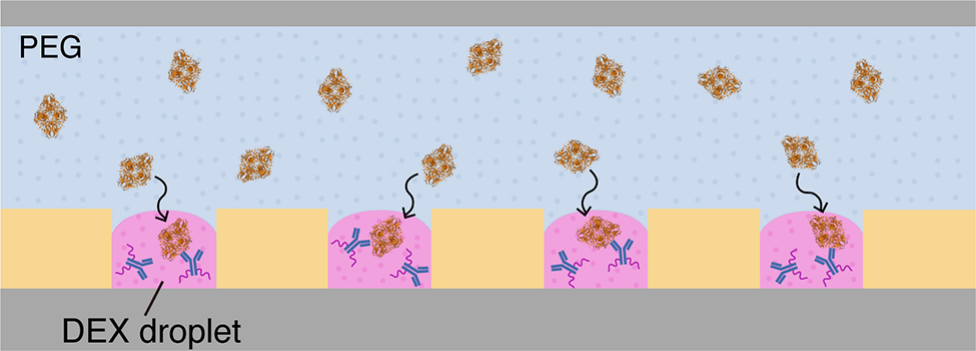

We developed an on-chip enrichment method based on an aqueous two-phase system of dextran/polyethylene glycol mix, DEX/PEG ATPS, for digital bioassay. Accordingly, we prepared an array device of femtoliter reactors that displays millions of uniformly shaped DEX-rich droplets under a PEG-rich medium. The DEX-rich droplets effectively enriched DNA molecules from the PEG-rich medium. To quantify the enrichment power of the system, we performed a digital bioassay of alkaline phosphatase (ALP). Upon genetically tagging ALP molecules with the DEX-binding domain (DBD) derived from dextransucrase, the ALP molecules were enriched 59-fold in the DEX droplets in comparison to that in a conventional digital bioassay. Subsequently, we performed a Cas13-based digital SARS-CoV-2 RNA detection assay to evaluate the performance of this system for a more practical assay. In this assay, the target RNA molecules bound to the DBD-tagged Cas13 molecules were effectively enriched in the DEX droplets. Consequently, an enrichment factor of 31 was achieved. Enrichment experiments for nonlabeled proteins were also performed to test the expandability of this technique. The model protein, nontagged β-galactosidase, was enriched in DEX droplets containing DBD-tagged antibody, with an enrichment factor of over 100. Thus, this system enabled effective on-chip enrichment of target molecules to enhance the detection sensitivity of digital bioassays without using external instruments or an external power source, which would be applicable for on-site bioassays or portable diagnostic tests.

Digital bioassay has emerged as a bioanalysis strategy that binarizes the signals from the microcompartments encapsulating no molecules or a single molecule of enzymes or enzyme-labeled molecules for quantification with single-molecule detection sensitivity.^1−4^ Although single-molecule enzymatic assay by micro-compartmentalization was reported in 1960s,^5^ quantitative digital bioassays have been available since microfabrication technology enabled the generation of uniformly shaped micron-sized compartments.^6,7^ Thus far, various formats have been reported for micro-compartmentalization, including w/o droplet arrays,^8,9^ w/o droplets generated by flow focusing,^10^ hydrogel particle templated droplets,^11^ slip chips,^12^ and uniform femto-liposomes.^13^ The widespread use of these micro-compartmentalization technologies has enabled the digitization of various enzyme assays, which allow the measurement of simple enzymes as well as nucleic acids, antigens, viruses, membrane transport transporters, etc. Thus, digital bioanalysis dramatically expands the range of assay items.^1−3,14^

One of the most prominent features of digital bioanalytical methods is its high detection sensitivity as well as high quantitativeness that facilitates the determination of the concentration of target molecules for several orders of magnitude. Therefore, certain digitized bioassays, e.g., digital enzyme-linked immunosorbent assay (digital ELISA), are expected to serve as future-generation diagnostic tests. In addition to the high sensitivity and quantitativeness, the digital bioassay promotes the investigation of molecule-to-molecule variation of enzyme activity that is masked in general ensemble measurements.^15−17^

The limit of detection (LOD) of digital bioassays is restricted by the total reactor volume, i.e., the total number of reactors multiplied by the volume of each reactor. Upon considering that three molecules are required to detect one or more positive reactors among all the reactors, with a detection probability of >95%,^4^ the practical LOD denotes the concentration corresponding to three molecules per total reactor volume. A simple strategy for the enhancement of the LOD is to increase the total number of reactors and/or the volume of reactors. However, the size and scale of micro-compartments exhibit certain physical constraints such as the device size of arrayed-type microreactor systems and the generation time for droplets with microdroplet devices.

Thus, off-chip enrichment processes are often employed in digital bioassays if the required detection sensitivity is higher than the theoretical LOD defined by the total reactor volume. In the case of digital ELISA with arrayed reactor systems,^18,19^ the immunocomplexes were formed on the microbeads and washed with buffer for B/F separation, followed by the suspension of beads in smaller aliquots than the initial sample volume, which resulted in the enrichment of target molecules. After enhancement with the off-chip enrichment process, digital ELISA achieved LOD values 10–100 times lower than the theoretical LOD values.^18,19^

Although such off-chip enrichment processes can readily improve the detection sensitivity, these methods require additional processes for solution handling and equipment such as centrifuges or solution dispensing systems.^20^ Therefore, the off-chip enrichment process poses one of the technical challenges for realizing compact digital bioassay systems suitable for on-site analysis such as diagnostic tests at home or in small clinics, although compact detection systems for digital bioassays were reported.^21,22^ Thus far, several methods were reported for on-chip enrichment that utilized dielectrophoresis or a magnetic field.^23−25^ These methods required external equipment such as magnets or an electric power source.

In this study, we conceived a method for on-chip enrichment based on an aqueous two-phase system (ATPS). In particular, we focused on the ATPS of dextran (DEX) and polyethylene glycol (PEG), because this system can preferentially partition nucleic acid polymers such as DNA,^26^ RNA,^27^ and certain proteins^28^ into DEX-rich phases (left-hand figure in Figure 1a). In this study, we initially developed the array system of uniformly shaped DEX-rich droplets, hereinafter termed DEX droplet for simplicity (right-hand figure in Figure 1a), based on a femtoliter reactor array device (FRAD) that was originally developed for conventional digital bioassays.^8^ In addition, we developed a tag system for partitioning proteins in a DEX-rich phase. Subsequently, we tested on-chip enrichment of tagged enzymes such as alkaline phosphatase or Cas13 proteins to develop a more sensitive digital bioassay beyond the theoretical LOD defined by the total reaction volume. We also confirmed the DEX droplet system enables on-chip enrichment of a nontagged protein when DEX droplets are preloaded with tagged antibody.

**Figure 1:**
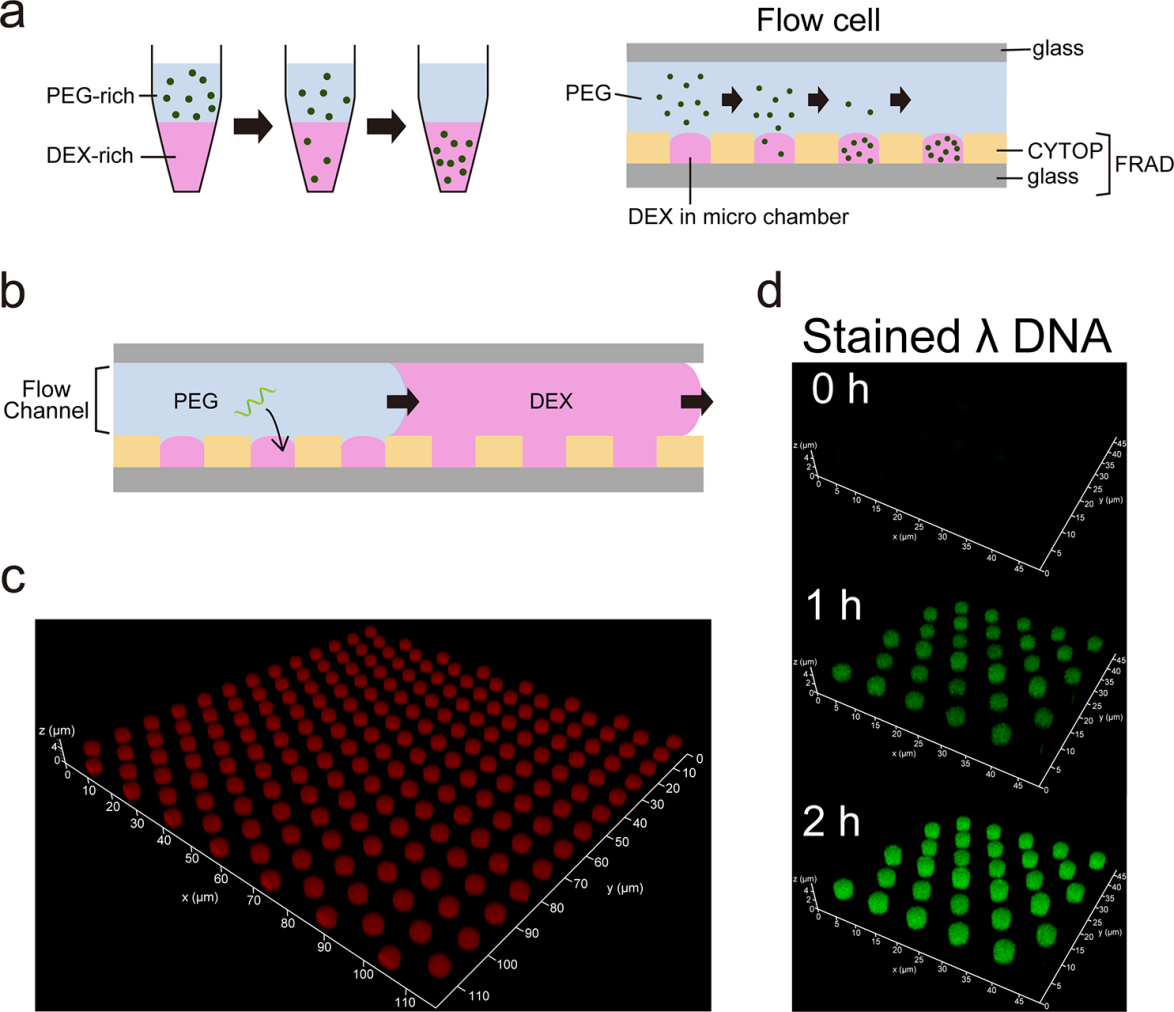
DEX droplet system. (a) Concept of enrichment of biomolecules with a DEX-rich phase of DEX/PEG ATPS in a conventional test tube (left) and with DEX droplets formed on a femtoliter reactor array device, FRAD (right). (b) Schematic of process of DEX droplet formation on a FRAD. (c) Confocal image of DEX droplets formed on a FRAD. DEX droplets were stained by spiking 0.1% (w/w) TRITC-DEX in 5.5% (w/w) DEX upon introducing DEX solution into the flow cell. (d) Time-lapse images of on-chip enrichment of fluorescently stained λ-DNA in DEX droplets.

## Results and Discussion

### Femtoliter DEX Droplet System

We employed a FRAD that displays a million micron-sized reactors (diameter: 4.4 μm; height: 3.2 μm) for the preparation of uniformly shaped DEX droplets with a volume of femtoliters. The size of the reactors is sufficiently small for a swift digital assay for enzymes and also sufficiently large for quantitative imaging with an optical microscope.^4^Figure 1b shows the preparation procedures; a DEX solution is initially infused into a flow cell with the FRAD located at the bottom to fill the reactors with the DEX solution. Thereafter, a PEG solution is introduced into the flow cell for flushing excess DEX solution. Consequently, DEX-rich droplets are formed in each micron-sized reactor of the FRAD under a PEG solution (Figure 1c). The ratio of the total reactor volume of the FRAD to the flow channel volume (Figure 1a and b) is 1:125. Thereby, the final concentration of DEX in the system is reduced to 1/126 when a PEG solution is introduced into a flow cell. In this study, we employed 5.5% (w/w) and 5.0% (w/w) for the initial concentrations of DEX and PEG solutions, respectively, considering the two conflicting requirements: sufficiently high to induce phase separation, but not too high to retain moderate viscosity for reproducible handling of the solution. The binodal curve determined in the present study ensures the phase separation at the final concentrations of DEX and PEG, 0.04% (w/w) and 5.0% (w/w), prepared by mixing 5.5% (w/w) DEX and 5.0% (w/w) PEG at a 1:125 ratio in a conventional tube (Figure S1). We determined the DEX concentration in the DEX-rich phase to be 4.9% (w/w). The formed DEX droplets exhibited high uniformity in volume: 73 ± 3.6 fL (*cv* = 4.9%) (Figure S2). Thus, the uniformly shaped DEX droplets with femtoliter volume were prepared following a simple procedure with a FRAD system.

We tested the capability of arrayed DEX droplets to enrich DNA molecules. According to a previous report on the ATPS of a DEX/PEG system,^26^ double-stranded DNA molecules of kbp length are effectively enriched in the DEX-rich phase. To test this phenomenon on the device, λ-DNA molecule with a 48 kbp length was introduced with 5% (w/w) PEG into the flow cell. As shown in Figure 1d, the DNA molecules were highly enriched with time, thereby exhibiting an efficient enrichment similar to the DEX-rich phase prepared in a test tube.

### Digital Bioassay of DBD-Tagged Alkaline Phosphatase with the DEX Droplet System

**Figure 2:**
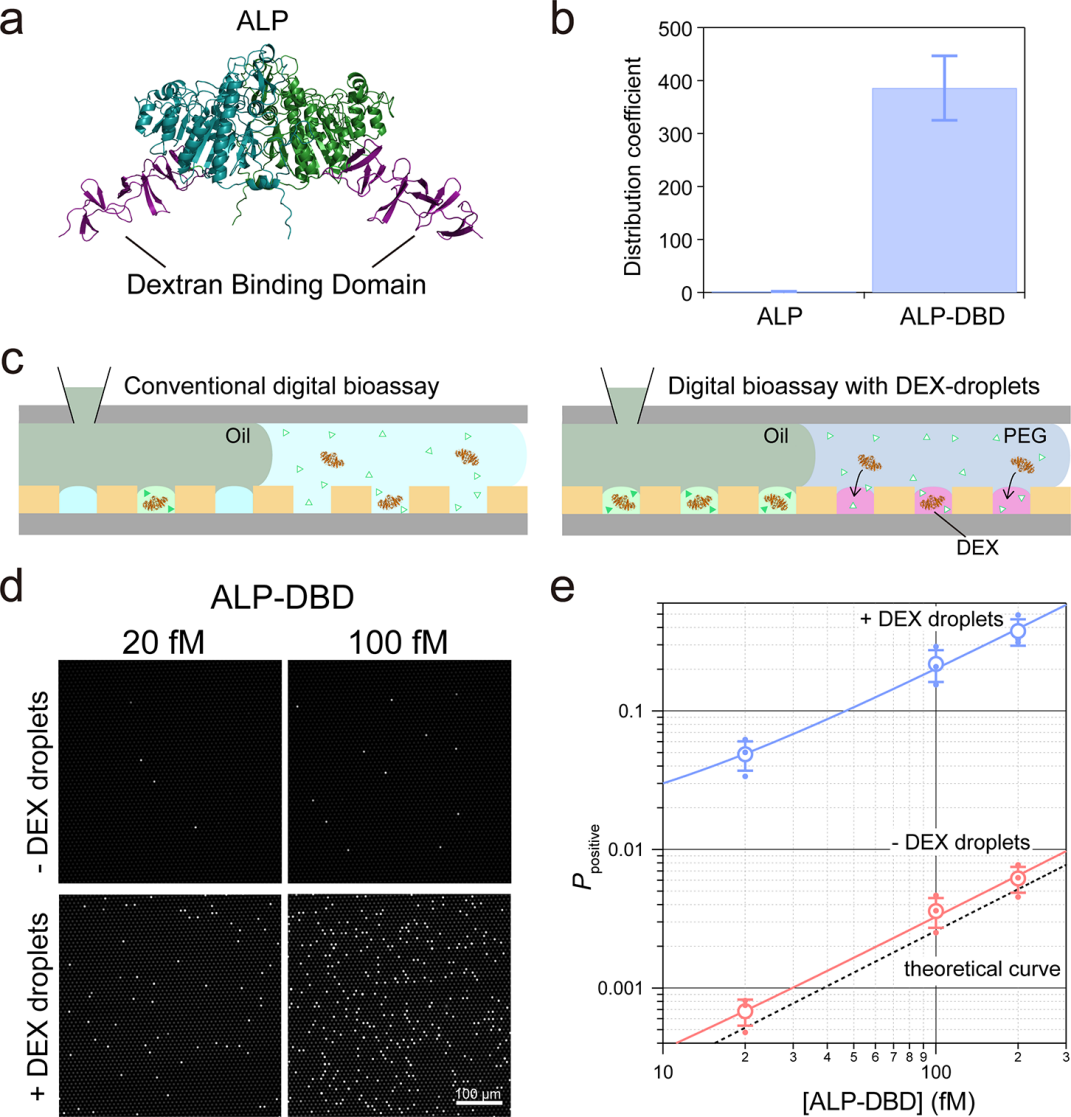
Digital bioassays of *Ec*ALP with the DEX droplet system. (a) ALP from *Escherichia coli* tagged with DBD at the C-terminus (ALP-DBD). The structure was modeled with alphafold. (b) Distribution coefficient (DC) of ALP or ALP-DBD in DEX/PEG ATPS. Error bars represent *s.d.* (*n* = 3). (c) Schematic of a conventional digital bioassay (left) and digital bioassay with DEX droplets (right). (d) Fluorescent images of a digital bioassay without DEX droplets (top) and with DEX droplets (bottom) at 20 fM, 100 fM ALP-DBD. (e) Probabilities of positive reactor (*P*_positive_) versus [ALP-DBD]: blue circles for *P*_positive_ of digital bioassays with DEX droplets and red ones for assays without DEX droplets. Blue and red open circles show the average of *P*_positive_ (*n* = 3), and error bars represent *s.d.* Broken line indicates theoretical value of *P*_positive_ from total reactor volume. Blue and red lines represent fitting to determine proportional coefficients against [ALP]: 1.9 × 10^–3^ [/fM] for the assay with DEX droplets and 3.2 × 10^–5^ [/fM] for assays without DEX droplets. The enrichment factor determined as a ratio of these coefficients was 59.

We investigated the availability of the DEX droplet system for a standard digital bioassay with alkaline phosphatase (ALP) from *Escherichia coli*, *Ec*ALP. Although DNA and RNA molecules are preferentially partitioned to the DEX-rich phase, several globular proteins are inefficiently enriched in the DEX-rich phase. For efficient enrichment of *Ec*ALP, we genetically fused *Ec*ALP with the DEX-binding domain (DBD) from *Leuconostoc mesenteroides* dextran sucrase (Figure 2a).^29^ DBD is a relatively small protein (14 kDa) with high affinity toward dextran (*K*_d_: 2.79 × 10^–9^ M).^30^ We measured the distribution coefficient (DC) of *Ec*ALP tagged with DBD (ALP-DBD) to the DEX-rich phase formed in a conventional test tube. ALP-DBD was mixed in DEX 0.04% (w/w)/PEG 5.0% (w/w) in a tube and centrifuged to separate the PEG-rich phase on the top and the DEX-rich phase at the bottom. Thereafter, aliquots from each phase were sampled for the measurement of ALP concentrations. To determine the *Ec*ALP concentration, we used a standard digital bioassay for ALP (refer to Figure S3). In principle, DC was determined as *C*_DEX_/*C*_PEG_, where *C*_DEX_ and *C*_PEG_ represent the concentrations of ALP-DBD in DEX- and PEG-rich phases, respectively. As depicted in Figure 2b, the DC of the intact *Ec*ALP was 1.9, whereas that of ALP-DBD was 385. Although intact *Ec*ALP is slightly prone to be enriched in the DEX-rich phase, the DBD tag enhances the partition by approximately 203 times. This signified that DBD enables the efficient partitioning of the tagged enzyme to DEX-rich phases. Note that *Ec*ALP is a homodimer enzyme, and therefore, ALP-DBD should include two DBDs.

We conducted a digital bioassay for ALP-DBD with the DEX droplet system. ALP-DBD molecules were spiked in 5.0% (w/w) PEG solution and then introduced to the flow cell to cover DEX droplets. After 10 min of incubation, an oil solution was introduced for sealing the DEX droplets (Figure 2c). At this instant, most of the enzyme molecules were enriched within 10 min (Figure S4). The typical fluorescence images obtained in the assays are portrayed in Figure 2d. For comparison, the assays without DEX droplets were performed as well. The digital bioassays with DEX droplets displayed evidently higher numbers of positive reactors at each concentration of ALP-DBD. The probabilities of positive reactors with and without DEX droplets at indicated concentrations of ALP-DBD are shown in Figure 2e. Although the data points without DEX droplets plummeted near the theoretical line estimated from the ALP-DBD concentration, those obtained with DEX droplets displayed evidently higher values. Data points were fitted with an equation consisting of a concentration-dependent term and an independent term. The enrichment ratio was then determined as the ratio of the concentration coefficients. The resultant enrichment factor of DEX droplets in comparison to assays without the DEX droplet system was 59. Thus, it was confirmed that the DEX droplet system efficiently enriches proteins labeled with DBD without any external instruments or external force.

### Cas13-Based Digital Bioassay for RNA Detection with the DEX Droplet System

We investigated the enrichment power of the DEX droplet system in a more practical form, a digital RNA counting assay. Cas13 is crRNA-guided RNase that exhibits trans-cleavage RNase activity, termed as collateral activity when Cas13 recognizes a target RNA via a complemental sequence on the crRNA preloaded on Cas13 protein. As the collateral activity of Cas13 is sufficiently high and readily detectable using self-quenched fluorescent reporter RNA, highly sensitive RNA detection methods were developed using Cas13 proteins.^31−33^ A Cas13-based digital bioassay for RNA detection is reported as well.^34,35^ For instance, a rapid Cas13-based digital bioassay was developed for detecting SARS-CoV-2 based on the FRAD system that displayed reactors with a 3 fL volume.^35^ This system enables a swift detection of SARS-CoV-2 RNA within a few minutes. However, the total reactor volume was correspondingly small, which consequently restricted the LOD under 10 fM unless the pull-down enrichment procedure with the magnetic beads and an external magnetic system was employed.^25^ Thus, the trade-off between the detection time and detection sensitivity is one of the technical challenges of Cas13-based digital bioassays. Here, we used the DEX droplet system in the digital detection of SARS-CoV-2 RNA for on-chip enrichment without using external instruments.

**Figure 3:**
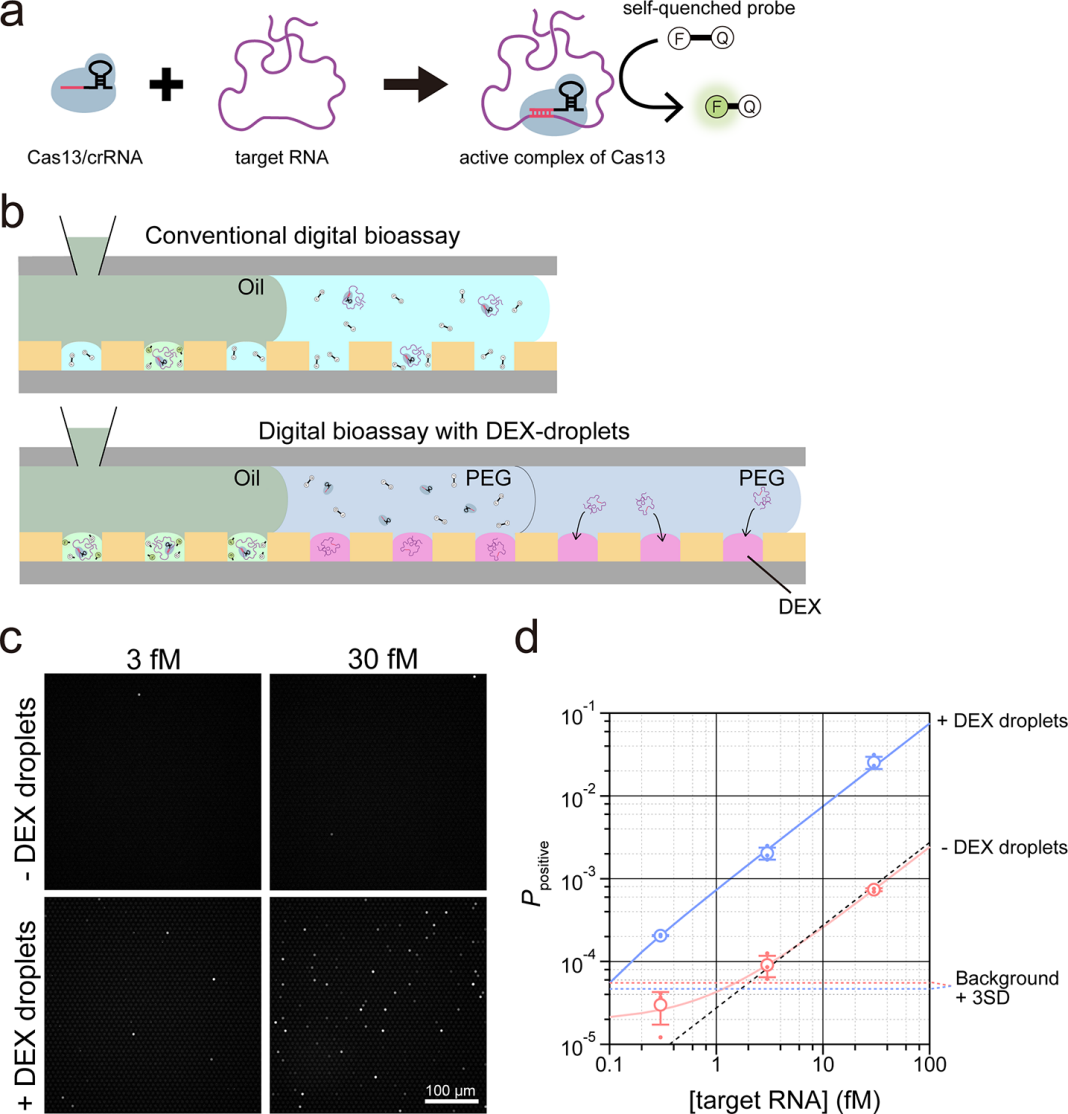
Digital RNA counting by Cas13 with the DEX droplet system. (a) Schematic of RNA detection using the Cas13 system. (b) Schematic of a conventional digital bioassay and digital bioassay with the DEX droplet system. In conventional methods, the Cas13/crRNA/target RNA complex and self-quenched probe are mixed and infused into the flow cell. In the assay with DEX droplets, the target RNA is enriched in the DEX droplets, and subsequently, Cas13/crRNA and the self-quenched probe are introduced into a flow cell. (c) Fluorescent images of digital RNA counting assays without (top) and with DEX droplets (bottom). (d) Probabilities of the positive reactor (*P*_positive_) versus target RNA concentration experimentally determined in assays with (blue) or without DEX droplets (red). The opened circles are the average of *P*_positive_ (*n* = 3), and error bars represent *s.d.* Black line displays theoretical *P*_positive_ estimated from total reactor volume. Blue and red solid lines display fitting curves to determine proportion coefficients against [target RNA]: 7.5 × 10^–4^ [/fM] for assays with DEX droplets and 2.4 × 10^–5^ for assays without DEX droplets. Enrichment factor, estimated as 31, represents the ratio of these coefficients. Blue and red dashed lines denote the limits of detection (LOD); the concentration corresponds to the mean +3 *s.d.* of the pseudopositive signal observed without target RNA.

Figure 3a shows the schematic of the Cas13-based assay for digital counting of RNA molecules encoding the SARS-CoV-2 S gene (3000 nt). For a fluorescent reporter substrate, we used a synthetic oligonucleotide carrying an AU sequence at the cleavage site for Cas13. The preliminary experiments posed two technical challenges: one is the pseudopositive signals observed even in the absence of the target RNA at a probability of ∼0.015%. As pseudopositive reactors increased the fluorescence with time, it could not be attributed to the fluorescence impurities in solution or on a device. Upon assuming that this was caused by the contamination of nonspecific ribonucleases from the samples or environment, we included another type of a reporter oligo nucleotide without recognition sequence for Cas13. The reporters with and without the recognition site for Cas13 were designed to produce green and red fluorescence, respectively. Thus, the dual reporter system facilitates the discrimination of the collateral activity of Cas13 as a green fluorescence signal from the contamination of a nonspecific ribonuclease that produces both the fluorescence signals. The dual reporter system suppressed the pseudopositive signal to approximately 40% (Figure S5). The origins of the remaining pseudopositives are unknown.

Another technical issue found in preliminary experiments was that the probability of the positive reactors was not as high as expected from the distribution coefficient of RNA into the DEX-rich phase. We hypothesized that although the target RNA molecules are once enriched in the DEX droplets, the outwardly exposed portions of the target RNA bound on Cas13 protein are digested by the activated Cas13, which releases the target RNA-Cas13 complex from DEX droplets. Based on this assumption, we tagged Cas13a using two repetitions of DBD. Consequently, the probability of the positive reactor significantly increased, thereby supporting the above assumption.

Digital RNA detection with and without DEX droplets was performed for comparison (Figure 3b). The fluorescence images obtained at 3 and 30 fM of the target RNA molecule are depicted in Figure 3c. Evidently, assays with DEX droplets detected more fluorescence reactors than those without DEX droplets. The probabilities of positive reactors, *P*_positive_, are plotted against the target RNA concentration (Figure 3d). In each assay with or without DEX droplets, the data points exhibited adequate linearity against the target concentration, excluding the data point at 0.3 fM without DEX droplets, where *P*_positive_ is relatively highly affected by pseudopositive reactors. Based on the linear fittings of the data points, the enrichment factor determined by DEX droplets was determined to be 31. Consequently, the LOD was 0.089 fM, which is lower than the theoretical LOD estimated as three molecules per the total reactor volume,^4^ 0.47 fM. Thus, the DEX droplet system allows the improvement of the LOD beyond theoretical limitation by its enrichment power.

### On-Chip Enrichment of Nontagged Enzyme with the DEX Droplet System

**Figure 4:**
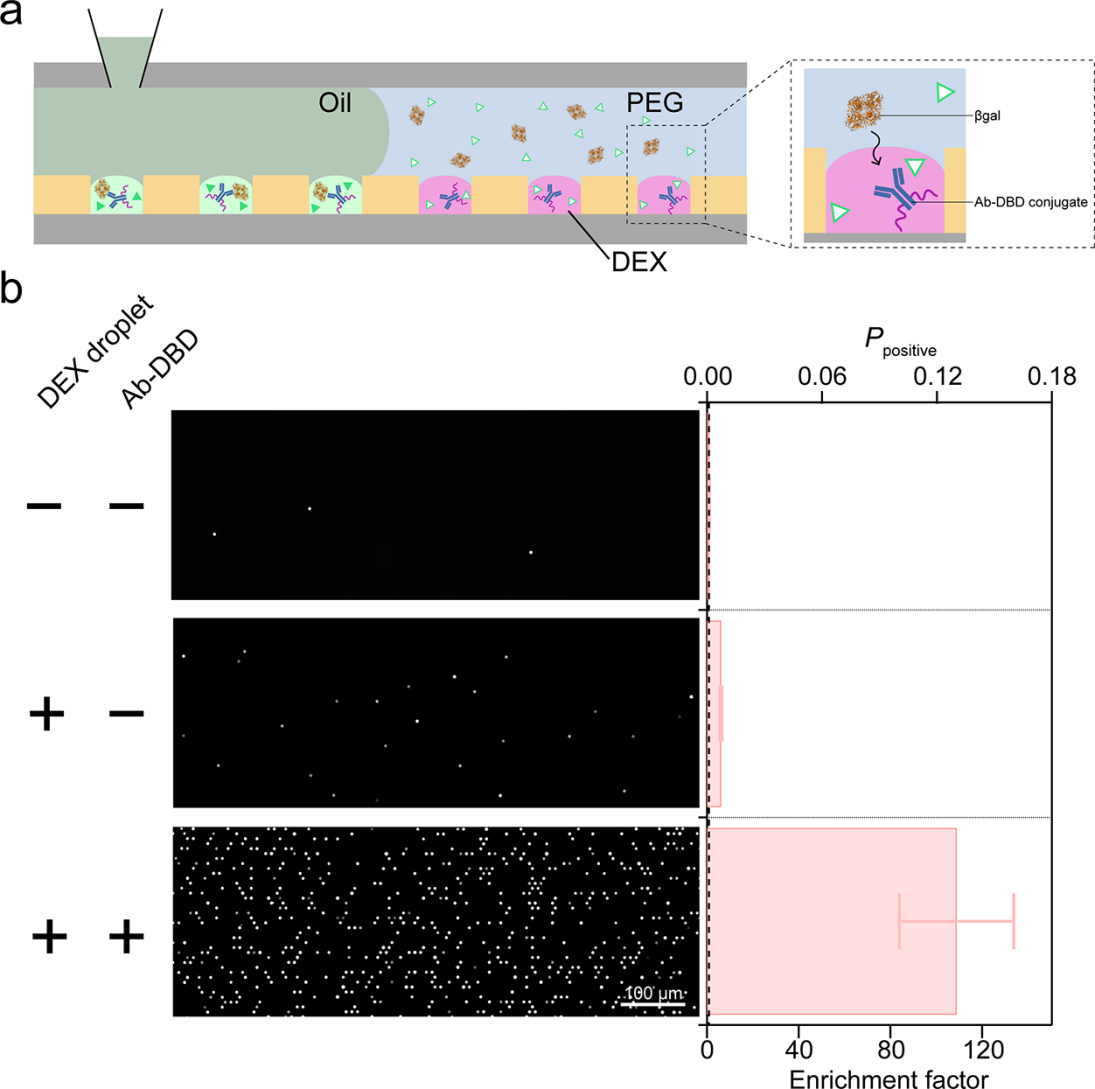
On-chip enrichment of nontagged enzyme with DEX droplets using Ab-DBD. (a) Schematic image of on-chip enrichment of β-galactosidase enzyme. For efficient capture of the enzyme, antibody molecules against β-galactosidase were loaded into DEX droplets, prior to the enzyme enrichment. (b) Fluorescent images of digital bioassays w/o DEX droplets and Ab-DBD (−, −), w/DEX droplets and w/o Ab-DBD (+, −), and w/DEX droplets and Ab-DBD (+, +). The right bar graph shows the counts of positive reactors (%), *P*_positive_, and the enrichment factors for the (−, −) condition: 6.0 for (+, −) and 108 for (+, +).

To demonstrate the expandability of the on-chip enrichment strategy to nontagged protein, we developed the DEX droplet system containing antibody tagged with DBD, Ab-DBD. As a model target protein, we employed β-galactosidase, a homotetramer enzyme. The IgY molecule against β-galactosidase was labeled with DBD to produce Ab-DBD and loaded to DEX droplets. β-Galactosidase molecules spiked in PEG solution were injected with a fluorogenic substrate into a flow cell chamber. After 10 min of incubation, DEX droplets were sealed with oil to conduct a digital bioassay of β-galactosidase (Figure 4a). We also conducted digital bioassays without DEX droplets and/or Ab-DBD for comparison purposes. Figure 4b shows the fluorescence images of the digital bioassays. Although the digital bioassay (w/DEX droplet and w/o Ab-DBD) showed a higher count of positive reactors against the conventional bioassay (w/o DEX droplets and w/o Ab-DBD), the assay w/DEX droplet and w/Ab-DBD showed a higher number of positive reactors. The resultant enrichment factor with DEX droplets and Ab-DBD was 108, very close to the theoretical maximum value, 125. This enrichment factor is even higher than those determined in DBD-ALP or DBD-Cas13 experiments. The higher enrichment power is attributable to the higher number of DBD in the complex of β-galactosidase and Ab-DBD, which would result in a higher distribution coefficient of the complex in DEX-rich phase compared with ALP-DBD and Cas13-DBD, of which the numbers of DBD are 2.

## Study Limitations

We note some limitations of this method. The system includes two major determinants for the enrichment factor: volume ratio of total reactor volume and flow cell volume and the distribution coefficient of DBD-tagged proteins. Although a straightforward strategy to enhance the enrichment factor is to increase the flow cell volume, it would compromise rapid enrichment because the enrichment process depends on the diffusion of target molecules, the time scale of which is proportional to the square of the thickness of the flow cell. Therefore, the serial buffer introduction would circumvent the exponential extension of the enrichment process. More importantly, the enhancement of the distribution coefficient of the DBD-tagged protein is a more beneficial strategy. In addition to introducing more repeats of DBD to enzymes, the development of DBD with higher affinity can be considered effective as well. However, the specific enrichment of proteins into DEX droplets requires tagging with DBD via genetic fusion or association with DBD-tagged proteins. In contrast, although the enrichment of DNA does not require tagging, sequence-specific enrichment is unavailable. Furthermore, molecules whose function is suppressed or inhibited in the DEX-rich phase are difficult to use in this system.

## Conclusions

This study established a method with the use of FRAD for the preparation of regularly shaped DEX droplets with volume ranging in femtoliters. Flow focused microfluidic systems were reported for the generation of monodisperse DEX droplets.^36−38^ However, to avoid spontaneous droplet fusion, the droplets were often solidified, compromising fluidity as well as the inner hydration of DEX droplets, which are crucial for the enrichment of biomolecules. As the DEX droplets prepared with the microfluidic systems were relatively large in size, the digital bioassay based on enzymatic activity is practically challenging. The femtoliter DEX droplets developed in this study were placed on the arrayed micron-sized cavity without solidification. Therefore, the droplets were observed to retain the intrinsic nature of a DEX-rich phase droplet.

By considering the intrinsic capability of DEX droplets to enrich biomolecules from the surrounding PEG-rich medium, this study performed the on-chip enrichment of DNA, RNA, and proteins and, subsequently, conducted digital bioassays. Under these conditions, an over 30-fold enrichment of the ALP molecules and Cas13/RNA complexes was achieved when the proteins were tagged with DBD. Consequently, Cas13 achieved a sub-femto-level molar detection in a digital RNA counting assay without using the off-chip enrichment procedures or pull-down methods with an external apparatus such as a magnetic system or electrodes. We also developed an on-chip enrichment strategy for nontagged proteins by use of DEX droplets preloaded with antibody tagged with DBD. As a result, we observed the highest enrichment factor, 108 for β-galactosidase. This experiment demonstrates the expandability of this system to nontagged proteins. It is expected that the DEX droplet system with DBD-tagged antibody allows highly sensitive digital detection of proteins in clinical samples by enabling specific on-chip enrichment of target protein from mixed samples. These features of the digital bioassay with DEX droplets will enable strategies for designing mobile systems with highly sensitive digital bioassays that are highly anticipated for the realization of transportable diagnostic systems in the home-care sector.

Furthermore, we developed the dual reporter system to reduce false-positive signals for highly sensitive digital RNA counting assay using the Cas13 protein. Potentially, the false-positive signals discriminable with the dual reporter system originated from nonspecific ribonuclease contaminated from the samples or environment. As ribonucleases are ubiquitously detected in biological samples, this method would be powerful for digital RNA counting assays for various types of clinical samples. Nonetheless, the origin of the remaining false-positive signals is unknown, and further investigations are required.

## Experimental Methods

### Chemicals

Polyethylene glycol (MW: 35 kDa), dextran from *Leuconostoc* spp. (MW: 450–650 kDa), TRITC-dextran (MW: 500 kDa), and tetrazine(Tz)-PEG5-NHS were purchased from Merck (Sigma-Aldrich), Germany. Fluorinert–FC40 and FC43 (3M, USA), fluorescein diphosphate (FDP) (AAT Bioquest, USA), SYBR Gold (Thermo Fisher Scientific, USA), SURFLON S-386 (AGC Seimi Chemical, Japan), BSA (New England Biolabs, USA), Fomblin Y-LVAC25/6 (Solvay, Belgium), fluorescein di-β-d-galactopyranoside (FDG) (Abcam, England), and *trans*-cyclooctene (TCO)-PEG4-NHS (TCI, Japan) were sourced from respective suppliers.

### Proteins and RNAs

A highly active mutant of ALP from *Escherichia coli*, ALP(D101S), was used throughout the experiments, which is termed as ALP or *Ec*ALP for simplicity. To prepare the ALP tagged with a dextran-binding domain from *Leuconostoc mesenteroides* dextransucrase^29^ (ALP-DBD), ALP was genetically fused with DBD at the C-terminus of ALP. The proteins of ALP or ALP-DBD were prepared in an *in vitro* TXTL system (PURExpress, New England Biolabs, USA) as reported earlier.^16^ In the digital RNA counting assays, we utilized Cas13a from *Leptotrichia wadei*. With minor modifications, Cas13 tagged with DBD was purified according to a previous report.^31^ The size exclusion chromatography was omitted, because DBD can adhere to the SEC column obtained from dextran. The target RNA for the digital RNA counting assay using Cas13 was designed to encode a fragment of the S gene of SARS-CoV-2 and to be 3000 nt for efficient enrichment in the DEX-rich phase. DNA encoding the designed RNA was synthesized by Eurofins Genomics, Japan. RNA was prepared from the synthesized DNA with a ScriptMax Thermo T7 transcription kit (TOYOBO, Japan) and a NucleoSpin RNA cleanup kit (Macherey-Nagel). crRNA was synthesized by Synthgo, US. β-Galactosidase (Wako, Japan) and anti-β-galactosidase IgY antibody (Abcam, England) were sourced from respective suppliers.

### Preparation of the Ab-DBD Conjugate

DBD was expressed overnight at 37 °C in Rosetta (DE3) cells, which harbored the plasmid coding for two DBD genes, where the His6-tag, twin-strep-tag, and SUMO-tag were inserted at the N-terminus of the DBD gene. The enzyme was purified at 4 °C through a nickel-nitrilotriacetic acid column (Ni-NTA superflow, Qiagen) using Ni-NTA binding/wash buffer (20 mM Tris-HCl, pH 7.5, 500 mM NaCl, 1 mM DTT, 30 mM imidazole) and elution buffer (Ni-NTA wash buffer supplemented with 300 mM imidazole). The elution was through a Strep-Tactin column (Strep-Tactin Superflow Plus, Qiagen) using Strep-Tactin wash buffer (20 mM Tris-HCl, pH 8.0, 500 mM NaCl, 1 mM DTT). SUMO protease solution (Strep-Tactin wash buffer supplemented with 20 nM SUMO protease and 0.15% (w/w) NP-40) is added to the resin, and the cleavage of the SUMO-tag is allowed to proceed overnight at 4 °C. The concentration of protein in the elution was determined by the absorbance at 280 nm.

To prepare the anti-β galactosidase IgY antibody (Ab)-DBD conjugate, the inverse electron-demand Diels–Alder cycloaddition between tetrazine (Tz) and TCO was utilized. The antibody and DBD were incubated with 40 molar equiv of TCO-PEG4-NHS and 2 molar equiv of Tz-PEG5-NHS for 1 h at RT, respectively. Unreacted TCO-PEG4-NHS and Tz-PEG5-NHS were removed using a 7 k Zeba spin desalting column. TCO-labeled antibody and Tz-labeled DBD were mixed with a molar ratio of 1:5 and incubated for 2 h at RT.

### Femtoliter Reactor Array Device

Following a previous study, the FRADs were prepared using photolithography microfabrication.^16^ Accordingly, the flow cells were assembled with a FRAD, a top glass with inlet and outlet holes, and using a double-sided tape (∼80 μm) as a spacer.^16^ The top glasses for the flow cell were precoated with CYTOP 809M to avoid the nonspecific binding of biomolecules.

### Microscopic Imaging

The confocal fluorescence images were acquired using a TCS SP8 X (Leica Microsystems, Germany), which is a laser scanning confocal microscope equipped with a white-light laser (Leica Microsystems, Germany). The fluorescent signals from TRITC-DEX were obtained using a HyD detector (Leica Microsystems, Germany). The confocal fluorescence images were analyzed using Fiji, an image processing package of ImageJ. Additionally, the epifluorescence images were obtained using an epifluorescence microscope (Eclipse Ti2, Nikon or Olympus IX83, Olympus Co.) equipped with a sCMOS camera (Zyla sCMOS or Andor neo, Andor Technology) and an LED light source (X-Cite Turbo or X-cite XYLIS, Excelitas Technologies). The epifluorescence images were acquired using Fiji (image processing package of ImageJ) and custom-written macros.

### Formation of Uniform DEX Droplets Using the FRAD

A solution of DEX at indicated concentrations of 5.5% (w/w) with 0.1% (w/w) TRITC DEX was infused into a flow cell. Subsequently, PEG at indicated concentrations (5.0% (w/w)) was infused to flush the excess amount of DEX solution from the flow channel. DEX solution remained within each micron-sized cavity on the FRAD, thereby forming a uniformly shaped DEX droplet. In DNA enrichment experiments, a DNA solution containing 12.5 ng/μL λ-DNA stained with 1× SYBR gold in 5.0% (w/w) PEG was infused into the flow cell.

### Quantification of the Distribution Coefficient of ALP-DBD

For separating the DEX-rich phase at the bottom and PEG-rich phase at the top, 0.04% (w/w) DEX, TRITC-DEX 0.001% (w/w), 5.0% (w/w) PEG, 0.5 nM ALP or ALP-DBD, and a reaction buffer for ALP (ALP buffer; 1 M diethanolamine, pH 9.25, 1 mM MgCl_2_, 0.02% (w/v), and Tween20) were appropriately mixed and centrifuged at 15 000 rpm for 3 min. Thereafter, small aliquots were sampled from each phase. The concentration of ALP in the samples was determined in a digital bioassay for ALP after dilution with the reaction mix for ALP (10 μM Alexa647 and 1 mM FDP, 1 M diethanolamine, pH 9.25, 1 mM MgCl_2_, and 0.02% (w/v) Tween20).

### Digital Bioassay of ALP-DBD with the DEX Droplet System

Prior to assay, the blocking solution (Tween20 0.2% (w/v)) was infused into a flow cell chamber to prevent the nonspecific binding of ALP molecules onto the flow cell surface. Thereafter, a mixture of 5.5% (w/w) DEX and 0.1% (w/w) TRITC-DEX in the ALP buffer was infused into the pretreated flow cell. ALP or ALP-DBD was introduced in the ALP buffer with 5.0% (w/w) PEG. After a 10 min incubation, the PEG solution was flushed out with FC40 oil, followed by the injection of fomblin oil to prevent evaporation.

### Digital RNA Counting by Cas13a with the DEX Droplet System

Self-quenched probes were prepared to distinguish the RNA cleavage reaction by Cas13 from nonspecific ribonucleases. Cas13 preferentially cleaved the AU, UU, CU, and GU dinucleotide motifs, but it exhibited low activity against the remaining motifs.^39^ Thus, FAM-AU-BHQ1 (5′-6FAM-taAUgc-BHQ1-3′) for Cas13 cleavage and Cy5-AC-BHQ3 (5′-Cy5-taACgc-BHQ3-3′) for nonspecific ribonuclease were procured from Fasmac, Japan. The capital and lowercase letters in the names of the probes represent ribo- or deoxyribonucleotide, respectively.

The procedure of digital RNA counting by Cas13 with DEX droplets is detailed as follows. First, a blocking solution containing 0.5 mg/mL BSA, 0.06% (w/w) S-386, 500 nM FAM-AU-BHQ1, and 500 nM Cy5-AC-BHQ3 in Cas13 buffer (20 mM HEPES-NaOH, pH 6.8, 60 mM NaCl, 6 mM MgCl_2_) was infused into a flow cell. Thereafter, DEX mix (5.5% (w/w) DEX and 0.1% (w/w) TRITC-DEX) in Cas13 buffer was infused into the flow cell, followed by flushing with a PEG mix (5% (w/w) PEG, 0.06% (w/w) S-386) in Cas13 buffer, which indicated the amount of target RNA. After incubation for 10 min, the PEG mix was infused with the following components: 45 nM Cas13, 22.5 nM crRNA, 3 μM FAM-AU-BHQ1, 10 μM Cy5-AU-BHQ3, 2 U/mlL RNase inhibitor, and 0.06% (w/w) S-386. Furthermore, a sealing oil (1:1 mixture of FC43 and fomblin Y-LVAC25/6) was introduced. After 30 min of incubation at room temperature, the fluorescent images were obtained with the epifluorescence microscope. We performed the digital bioassay without DEX droplets by infusing the reaction mix (indicated amount of target RNA, 45 nM Cas13, 22.5 nM crRNA, 3 μM FAM-AU-BHQ1, 10 μM Cy5-AU-BHQ3, 2 U/mL RNase inhibitor, and 0.06% S-386 in Cas13 buffer) after the injection of the blocking solution. Ultimately, a sealing oil (1:1 mixture of FC43 and fomblin Y-LVAC25/6) was infused into the flow cell.

### Digital Bioassay of Nontagged β-Galactosidase with DEX Droplets Using Ab-DBD

First, the blocking solution was infused into a flow cell. Thereafter, a DEX mix in β-gal assay buffer (41 mM Na_2_HPO_4_, 0.74 mM KH_2_PO_4_, NaCl 68.5 mM, 0.01% (w/w) S-386) was infused into the flow cell, followed by flushing with a PEG mix in β-gal assay buffer containing 1 nM Ab-DBD. After incubation for 10 min for enrichment of the Ab-DBD conjugate from the PEG-rich phase to the DEX reactor, a PEG mix containing 33 fM β-galactosidase and 1 mM FDG in a β-gal assay buffer was infused. After a 10 min incubation, the PEG solution was flushed out with FC40 oil, followed by the injection of fomblin oil to prevent evaporation.

## Supporting Information Available

Binodal curve and estimated tie line for DEX/PEG APTS; diameter and volume of DEX reactors; estimation of distribution coefficient of ALP and ALP-DBD to ATPS for DEX/PEG; dependence of enrichment of ALP-DBD on incubation period; dual reporter system to suppress pseudopositive signal; model of enrichment factor for digital assay with DEX reactors (PDF)
