# Supplementary material for: On-Chip Enrichment System for Digital Bioassay Based on Aqueous Two-Phase System
Source: ACS Nano. 2022 Dec 29;17(1):212–20. doi: 10.1021/acsnano.2c06007 (PMC9835982; doi:10.1021/acsnano.2c06007)
Supplement: Supplementary file 1 — nn2c06007_si_001.pdf [file nn2c06007_si_001.pdf]

On-chip enrichment system for digital bioassay based on aqueous two-phase system

Yoshihiro Minagawa[1], *Shoki Nakata*[1], *Motoki Date*[1], *Yutaro Ii*[1], *Hiroyuki Noji*[1 *]

[1] Department of Applied Chemistry, The University of Tokyo, 7-3-1 Hongo, Bunkyo-ku, 113-8656, Japan.

Table of Contents

Figure S1 2
Figure S2 3
Figure S3 4
Figure S4 5
Figure S5 6
Figure S6 7
Supplementary Table 1 8

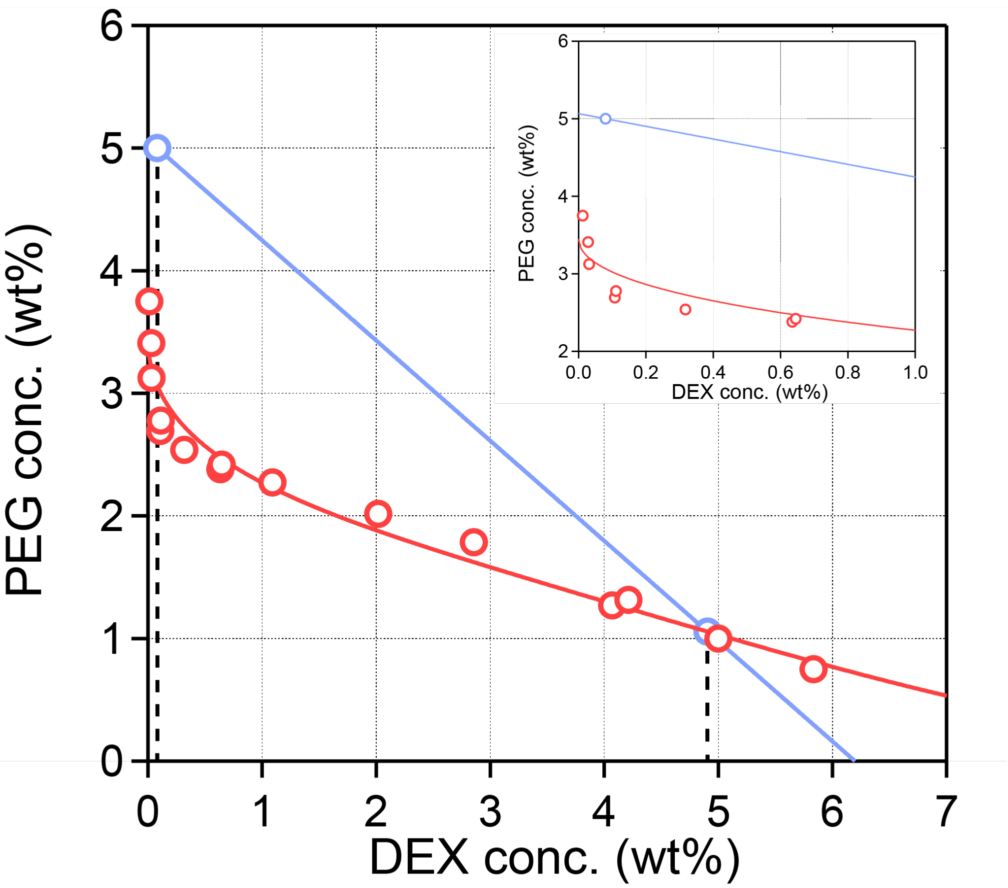

Figure S1 Binodal curve and estimated tie line.

The red circles show the dilution point at which DEX/PEG ATPS turns into a miscible solution, which is determined by the titration method.[1] The red line shows the result of fitting with equation: $[PEG] = C_1\exp(C_2[DEX]^{0.5} + C_3[DEX]^3)$.[2, 3] The blue circles show the concentration of mixing 5.5%(w/w) DEX and 5.0%(w/w) PEG at 1:125 ratio containing 0.03%(w/w) TRITC-DEX and intersection with the binodal curve at the concentration of DEX-rich phase (4.9%(w/w)) after mixing. The blue line show tie-line by connecting two blue circles.

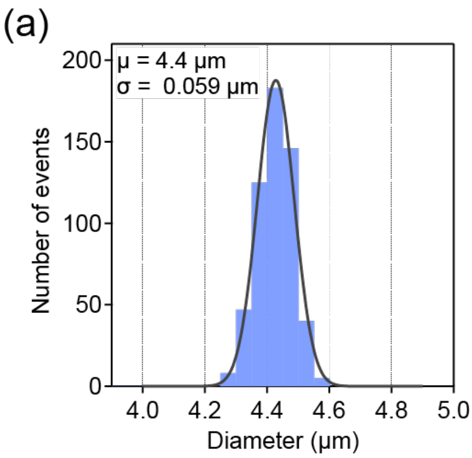

Figure S2 Diameter and volume of DEX reactors formed in femtoliter chamber array.

(a) Diameter and (b) volume of DEX reactors obtained by analyzing confocal microscope images of TRITC-DEX fluorescence in Figure 1. μ and σ in top-left of figures represent mean and standard deviation obtained by Gaussian fitting.

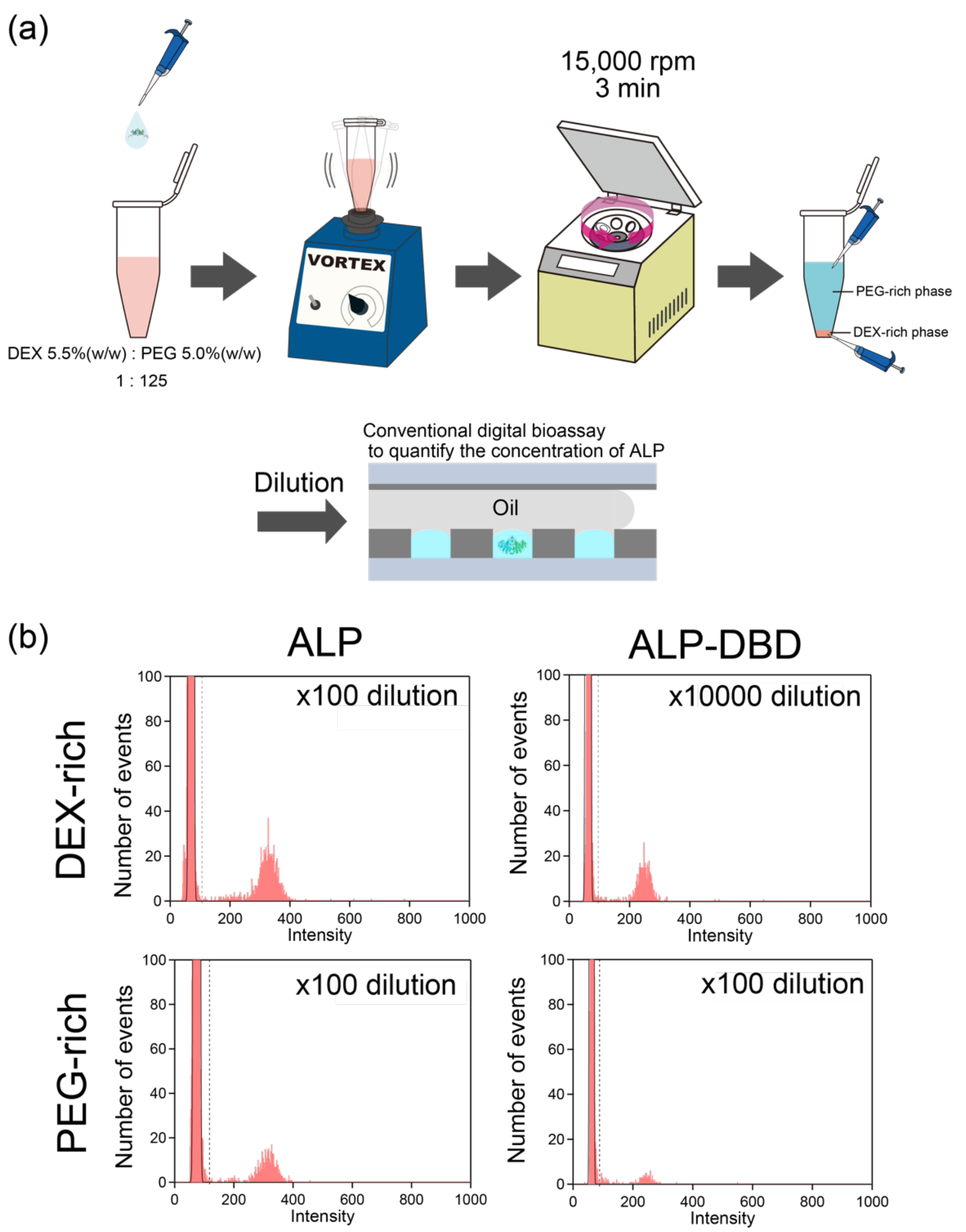

Figure S3 Distribution coefficient of ALP and ALP-DBD to ATPS for DEX/PEG.

(a) Schematic of experimental protocol for estimating distribution coefficient. (b) Histogram of fluorescence intensity by fluorescein, the product of ALP hydrolyzing FDP. Top-left and bottom-left display histograms for ALP in collected DEX- and PEG-rich phases. Collected DEX- and PEG-rich phases were diluted 100-fold infused into a femtoliter chamber array device such that the average number of enzymes per reactor is less than 1. Top-right and top-left display histograms of ALP-DBD in DEX- and PEG-rich phases, which were diluted 10000-fold and 100-fold, respectively.

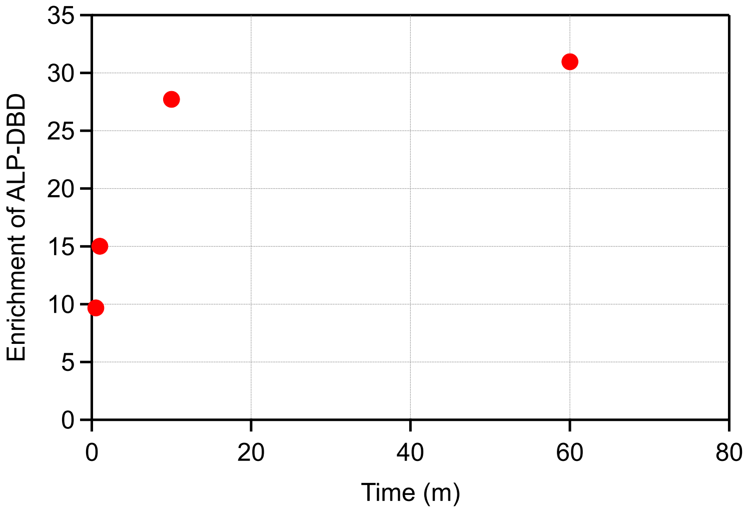

Figure S4. Dependence of enrichment of ALP-DBD on incubation period.

Time dependence of enrichment of ALP-DBD in a digital bioassay with DEX droplets. Incubation period represents time from infusion of PEG containing 400 fM ALP-DBD to flushing out with FC40. Enrichment factor was determined based on ratio of probability of positive reactors.

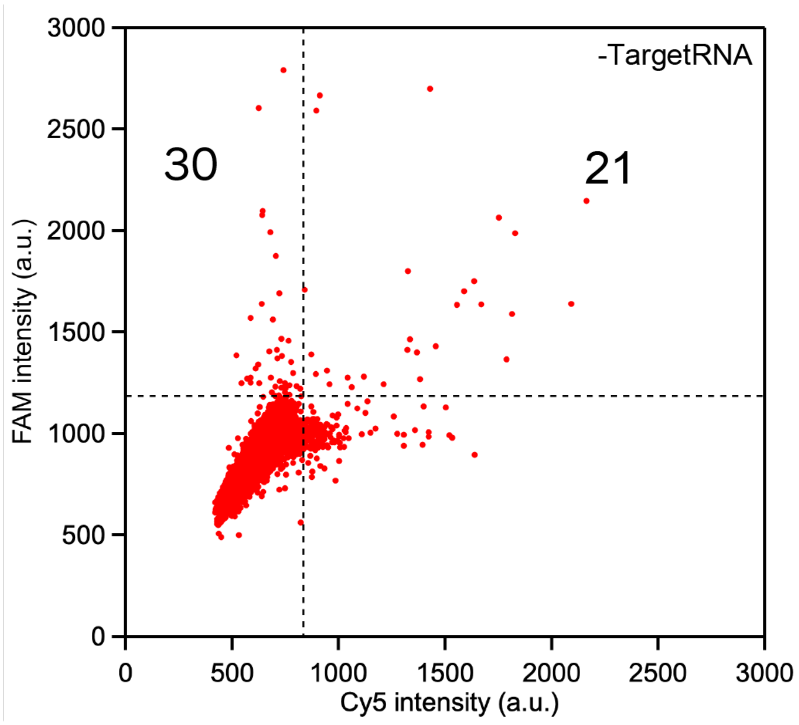

Figure S5. Dual reporter system to suppress pseudo-positive signal.

The reporters with and without the recognition site for Cas13 were designed to produce FAM and Cy5 fluorescence, respectively. Fluorescence intensity of FAM and Cy5 obtained from Cas13-based digital bioassay in the absence of the target RNA was plotted in two dimensions. Reactors emitting FAM fluorescence above a threshold value (horizontal dashed line) were pseudo-positive signals. In low-concentration target RNA conditions, these pseudo-positive reactors have a relatively strong effect on P_{positive}. Among them, those with Cy5 fluorescence intensity above a threshold can be excluded as pseudo-signal in the analysis.

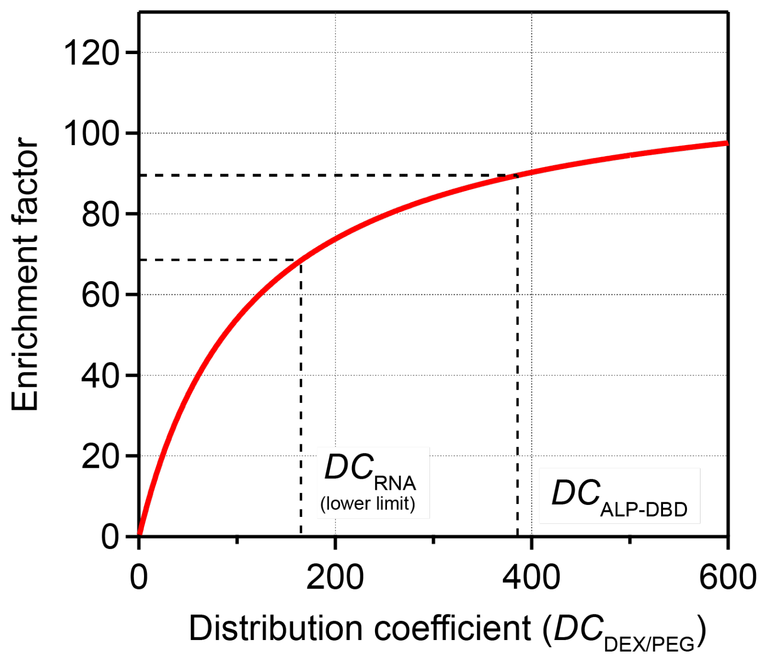

Figure S6. Model of enrichment factor for digital assay with DEX reactors.

Herein, we modeled the relationship between the enrichment factor in digital bioassay with DEX droplets and distribution coefficient in a tube. The distribution coefficients obtained in the tube are given by

$$DC_{DEX/PEG} = \frac{C_{DEX}}{C_{PEG}},$$

where C_{DEX} and C_{PEG} represent the concentration of target in DEX- and PEG-rich phases. As the number of molecules in the solution is conserved.

$$C_{PEG} \cdot V_{PEG} + C_{DEX} \cdot V_{DEX} = C_0 \cdot V_0,$$

where C_0 represents the initial concentration of target. V_0, V_{DEX}, and V_{PEG} represent the total volume, and the volume of DEX in micro chambers and PEG-rich phase in flow channel (Fig. 1a, 1b), respectively. The enrichment factor can be expressed as C_{DEX}/C_0.

$$enrichement = \frac{C_{DEX}}{C_0} = \frac{\frac{V_0}{V_{PEG}}}{\frac{C_{PEG}}{C_{DEX}} + \frac{V_{DEX}}{V_{PEG}}} = \frac{\frac{V_0}{V_{PEG}}}{\frac{1}{DC_{DEX/PEG}} + \frac{V_{DEX}}{V_{PEG}}}$$

Here, the volume ratio of PEG to DEX is defined as

$$R_{DEX/PEG} = \frac{V_{DEX}}{V_{PEG}}.$$

$R_{DEX/PEG}$ is 125 in digital bioassay with DEX droplets, indicating V_{PEG} is much larger than V_{DEX}. Thus, this can be expressed as $V_0 \fallingdotseq V_{PEG}$. Conclusively, the enrichment factor can be expressed as follows:

$$enrichement = \frac{1}{\left(DC_{DEX/PEG}\right)^{-1} + R_{DEX/PEG}}.$$

The distribution coefficient for ALP-DBD ($DC_{ALP\text{-}DBD}$) in the tube was 385 and that for the RNA (DC_{RNA}) was estimated as at least 164. The enrichment factor of ALP-DBD and digital Cas13 measurements estimated from the model were 90 and 68, which were higher than the experimental values.

Supplementary Table 1 RNA and self-quenched probe sequences used in this study

Target RNA	GGGUAACAUCACUAGGUUUCAAACUUUACUUGCUUUACAUAGAAGUUAUUUGACUCCUGGU<br>GAUUCUUCUUCAGGUUGGACAGCUGGUGCUGCAGCUUAUUAUGUGGGUUAUCUUCAACCUA<br>GGGAGAACAGCAAGAAGCACGAGAAGUACAAGAUCCGCGAGUACUAUCACAAGAUCAUCGG<br>CCGGAAGAACGACAAAGAGAACUUCGCCAAGAUUAUCUACGAAGAGAUCCAGAACGUGAAC<br>AACAUCAAAGAGCUGAUUGAGAAGAUCCCCGACAUGUCUGAGCUGAAGAAAAGCCAGGUGU<br>UCUACAAGUACUACCUGGACAAAGAGGAACUGAACGACAAGAAUAUUAAGUACGCCUUCUG<br>CCACUUCGUGGAAAUCGAGAUGUCCCAGCUGCUGAAAAACUACGUGUACAAGCGGCUGAGC<br>AACAUCAGCAACGAUAAGAUCAAGCGGAUCUUCGAGUACCAGAAUCUGAAAAAGCUGAUCG<br>AAAACAAACUGCUGAACAAGCUGGACACCUACGUGCGGAACUGCGGCAAGUACAACUACUA<br>UCUGCAAGUGGGCGAGAUCGCCACCUCCGACUUUAUCGCCCGGAACCGGCAGAACGAGGCC<br>UUCCUGAGAAACAUCAUCGGCGUGUCCAGCGUGGCCUACUUCAGCCUGAGGAACAUCCUGG<br>AAACCGAGAACGAGAACGAUAUCACCGGCCGGAUGCGGGGCAAGACCGUGAAGAACAACAA<br>GGGCGAAGAGAAAUACGUGUCCGGCGAGGUGGACAAGAUCUACAAUGAGAACAAGCAGAAC<br>GAAGUGAAAGAAAAUCUGAAGAUGUUCUACAGCUACGACUUCAACAUGGACAACAAGAACG<br>AGAUCGAGGACUUCUUCGCCAACAUCGACGAGGCCAUCAGCAGCAUCAGACACGGCAUCGU<br>GCACUUCAACCUGGAACUGGAAGGCAAGGACAUCUUCGCCUUCAAGAAUAUCGCCCCCAGC<br>GAGAUCUCCAAGAAGAUGUUUCAGAACGAAAUCAACGAAAAGAAGCUGAAGCUGAAAAUCU<br>UCAAGCAGCUGAACAGCGCCAACGUGUUCAACUACUACGAGAAGGAUGUGAUCAUCAAGUA<br>CCUGAAGAAUACCAAGUUCAACUUCGUGAACAAAAACAUCCCCUUCGUGCCCAGCUUCACC<br>AAGCUGUACAACAAGAUUGAGGACCUGCGGAAUACCCUGAAGUUUUUUGGAGCGUGCCCA<br>AGGACAAAGAAGAGAAGGACGCCCAGAUCUACCUGCUGAAGAAUAUCUACUACGGCGAGUU<br>CCUGAACAAGUUCGUGAAAAACUCCAAGGUGUUCUUUAAGAUCACCAAUGAAGUGAUCAAG<br>AUUAACAAGCAGCGGAACCAGAAAACCGGCCACUACAAGUAUCAGAAGUUCGAGAACAUCG<br>AGAAAACCGUGCCCGUGGAAUACCUGGCCAUCAUCCAGAGCAGAGAGAUGAUCAACAACCA<br>GGACAAAGAGGAAAAGAAUACCUACAUCGACUUUAUUCAGCAGAUUUUCCUGAAGGGCUUC<br>AUCGACUACCUGAACAAGAACAAUCUGAAGUAUAUCGAGAGCAACAACAACAAUGACAACA<br>ACGACAUCUUCUCCAAGAUCAAGAUCAAAAAGGAUAACAAAGAGAAGUACGACAAGAUCCU<br>GAAGAACUAUGAGAAGCACAAUCGGAACAAAGAAAUCCCUCACGAGAUCAAUGAGUUCGUG<br>CGCGAGAUCAAGCUGGGGAAGAUUCUGAAGUACACCGAGAAUCUGAACAUGUUUUACCUGA<br>UCCUGAAGCUGCUGAACCACAAAGAGCUGACCAACCUGAAGGGCAGCCUGGAAAAGUACCA<br>GUCCGCCAACAAAGAAGAAACCUUCAGCGACGAGCUGGAACUGAUCAACCUGCUGAACCUG<br>GACAACAACAGAGUGACCGAGGACUUCGAGCUGGAAGCCAACGAGAUCGGCAAGUUCCUGG<br>ACUUCAACGAAAACAAAAUCAAGGACCGGAAAGAGCUGAAAAAGUUCGACACCAACAAGAU<br>CUAUUUCGACGGCGAGAACAUCAUCAAGCACCGGGCCUUCUACAAUAUCAAGAAAUACGGC<br>AUGCUGAAUCUGCUGGAAAAGAUCGCCGAUAAGGCCAAGUAUAAGAUCAGCCUGAAAGAAC<br>UGAAAGAGUACAGCAACAAGAAGAAUGAGAUUGAAAAGAACUACACCAUGCAGCAGAACCU<br>GCACCGGAAGUACGCCAGACCCAAGAAGGACGAAAAGUUCAACGACGAGGACUACAAAGAG<br>UAUGAGAAGGCCAUCGGCAACAUCCAGAAGUACACCCACCUGAAGAACAAGGUGGAAUUCA<br>AUGAGCUGAACCUGCUGCAGGGCCUGCUGCUGAAGAUCCUGCACCGGCUCGUGGGCUACAC

	CAGCAUCUGGGAGCGGGACCUGAGAUUCCGGCUGAAGGGCGAGUUUCCCGAGAACCACUAC AUCGAGGAAAUUUUCAAUUUCGACAACUCCAAGAAUGUGAAGUACAAAAGCGGCCAGAUCG UGGAAAAGUAUAUCAACUUCUACAAAGAACUGUACAAGGACAAUGUGGAAAAGCGGAGCA UCUACUCCGACAAGAAAGUGAAGAAACUGAAGCAGGAAAAAAAGGACCUGUACAUCCGGAA CUACAUUGCCCACUUCAACUACAUCCCCCACGCCGAGAUUAGCCUGCUGGAAGUGCUGGAA AACCUGCGGAAGCUGCUGUCCUACGACCGGAAGCUGAAGAACGCCAUCAUGAAGUCCAUCG UGGACAUUCUGAAAGAAUACGGCUUCGUGGCCACCUUCAAGAUCGGCGCUGACAAGAAGAU CGAAAUCCAGACCCUGGAAUCAGAGAAGAUCGUGCACCUGAAGAAUCUGAAGAAAAAGAAA CUGAUGACCGACCGGAACAGCGAGGAACUGUGCGAACUCGUGAAAGUCAUGUUCGAGUACA AGGCCCUGGAAUAAGCGGCCGCACUCGAGGCCCGAAAGGAAGCUGAGUUGGCUGCUGCCAC CGCUGAGCAAUAA
crRNA	GAUUUAGACUACCCCAAAAACGAAGGGGACUAAAACGCAGCACCAGCUGUCCAACCUGAAG AAG
FAM-AU-BHQ1	6FAM-taAUgc-BHQ1*
Cy5-AC-BHQ3	Cy5-taACgc-BHQ3*

* Capital and lowercase letters represent RNA and DNA, respectively

Reference

1. Hatti-Kaul, R., Aqueous Two-Phase Systems: Methods and Protocols. *Springer* **2000**.
2. Merchuk, J. C.; Andrews, B. A.; Asenjo, J. A., Aqueous two-phase systems for protein separation. Studies on phase inversion. *J Chromatogr B Biomed Sci Appl* **1998,** *711* (1-2), 285-93.
3. Atefi, E.; Fyffe, D.; Kaylan, K. B.; Tavana, H., Characterization of Aqueous Two-Phase Systems from Volume and Density Measurements. *J Chem Eng Data* **2016,** *61* (4), 1531-1539.
